# Substantial Deep‐Soil Carbon Losses Outweigh Topsoil Gains in European Beech Forests Since the 1980s

**DOI:** 10.1111/gcb.70446

**Published:** 2025-09-01

**Authors:** Mathias Mayer, Klaus Dolschak, Emilia Winter Artusio, Michael Grabner, Michael Tatzber, Iftekhar U. Ahmed, Elisabeth Wächter, Selina Türtscher, Leopold Lindebner, Isolde K. Berger, Pétra Berger, Wolfgang Wanek, Torsten W. Berger

**Affiliations:** ^1^ Institute of Forest Ecology, Department of Ecosystem Management, Climate and Biodiversity BOKU University Vienna Austria; ^2^ Forest Soils and Biogeochemistry Swiss Federal Institute for Forest, Snow and Landscape Research (WSL) Birmensdorf Switzerland; ^3^ Institute of Wood Technology and Renewable Materials, Department of Natural Sciences and Sustainable Resources BOKU University Tulln an der Donau Austria; ^4^ Austrian Research Centre for Forests (BFW), Department of Forest Protection Vienna Austria; ^5^ Centre for Microbiology and Environmental Systems Science, Division of Terrestrial Ecosystem Research University of Vienna Vienna Austria

**Keywords:** climate change, ecosystem carbon, soil inventory, soil nutrients, soil organic matter, subsoil

## Abstract

Soils are a major reservoir for organic carbon (C), with subsoils (> 20–30 cm soil depth) storing most of this C. Predicting the response of deep‐soil C to global change remains a critical research priority; yet long‐term field observations for forests are scarce. In this study, we assessed decadal C dynamics in mineral soils to 90 cm depth of 62 temperate mature stands of European beech ( *Fagus sylvatica* ) in Austria using data from sampling campaigns in 1984, 2012, and 2022. Our results show an increase in C stocks between 0 and 20 cm and a significant decrease in C stocks at 20–50 cm and 50–90 cm soil depth, suggesting substantial C losses from deep soils. These deep soil C losses outweighed the C gain in topsoils, resulting in a soil C loss of −0.44 ± 0.19 Mg C ha^−1^ year^−1^ since the 1980s. Organic‐rich calcareous soils appeared to be particularly vulnerable to C loss, while soils containing high amounts of iron and manganese were less affected, probably because they stabilize C more effectively. We suggest that changes in regional climate (i.e., warmer and wetter) and factors such as changes in litter inputs and rooting depth may underlie the observed patterns of depth‐dependent soil C changes. The estimated soil C loss accounted for 17% of the C accumulated in aboveground biomass over the same period, as determined by dendrochronological analysis, indicating a reduction in the ecosystem's C sink capacity since the 1980s. Our results highlight the importance of including deep‐soil C storage in forest ecosystem C cycle assessments, as it plays a key role in the overall ecosystem C balance.

## Graphical abstract

Forest soils store large amounts of carbon, especially in deeper layers. Using long‐term data from European beech forests in Austria, we found that while topsoil carbon increased over four decades, deeper soils lost significant amounts of carbon. These losses outweighed the gains in topsoil, reducing the forest's overall carbon sink capacity. Our findings show that deep‐soil carbon is vulnerable to environmental change and must be considered when assessing forest carbon dynamics.

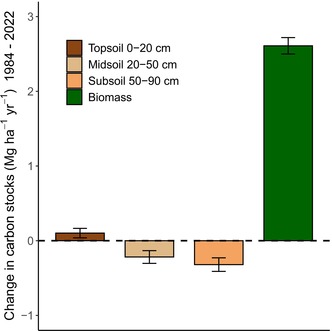

## 1 Introduction

Globally, soils store about 1500 Pg of organic carbon (C) in the first meter, with most of the C located below 20 cm depth (Jobbágy and Jackson 2000; Scharlemann et al. 2014). This deep‐soil C is typically older than topsoil C, indicating stabilization over centuries to millennia (Shi et al. 2020; Sierra et al. 2024). While deep soils may act as a critical C sink, they could also be vulnerable to losses under global climatic changes. For example, soils are projected to warm by approximately +2.3°C to 4.5°C at 1‐m depth by the end of the 21st century (Soong et al. 2020). This temperature increase could accelerate microbial decomposition and facilitate deep‐soil C loss, potentially increasing atmospheric CO_2_ concentrations (Hicks Pries et al. 2023).

Conversely, warming and rising CO_2_ levels and nitrogen (N) deposition may stimulate plant growth and ecosystem productivity, leading to increased litter inputs that could partially offset or even exceed these losses by supplying fresh organic matter to soils (Liu et al. 2005; Pretzsch et al. 2014; Kim et al. 2020; Gao et al. 2023). However, these organic inputs could also enhance microbial activity—not only in the topsoil but also in deeper horizons—through vertical transport of dissolved organic C and potential rhizosphere priming effects, where labile C inputs accelerate the decomposition of older soil organic matter (Fontaine et al. 2007; Chen et al. 2014; Jackson et al. 2019). In addition, global rainfall patterns are changing, including decreased or increased precipitation amounts, as well as intensified drought and rainfall events (Adler et al. 2020), all of which can affect microbial decomposition, plant growth, and overall C cycling (Frank et al. 2015). Long‐term declines in sulfur (S) deposition following major reductions in acidifying emissions in many regions may have contributed to improved conditions for plant growth. These changes can affect soil pH and nutrient dynamics, with potential consequences for plant nutrient status, microbial activity, and C storage (Berger et al. 2016). At the same time, the activity of microbial communities is modulated by mineralogical and chemical properties such as iron or calcium content, which can promote organo‐mineral associations that stabilize C (Lehmann and Kleber 2015a; Rowley et al. 2018). The balance between these competing C source and sink processes under future climate scenarios will ultimately shape changes in deep‐soil C stocks.

Our current understanding of the response of deep‐soil C to climate change relies mainly on modeling and a few whole‐profile soil warming experiments (Nottingham et al. 2020; Soong et al. 2021; Wang et al. 2022; Chen et al. 2024; Sierra et al. 2024). For example, a global modeling study showed that a 1°C increase in air temperature leads to a 5% reduction in deep‐soil C stocks, with an additional 2% loss for each additional 1°C of warming (Wang et al. 2022). Greater sensitivity was demonstrated in an experimental short‐term (< 5 years) warming study of the entire soil profile by 4°C, which increased soil CO_2_ losses by 26% to 55% compared to ambient soils (Nottingham et al. 2020; Soong et al. 2021; Chen et al. 2024).

In contrast, the temporal dynamics of deep‐soil C on time scales > 20 years remain poorly understood, as long‐term studies of deep soil C storage are scarce, especially in forest ecosystems (Harrison et al. 2011; Schrumpf et al. 2011; Grüneberg et al. 2019; Button et al. 2022; Hicks Pries et al. 2023). In one of the few studies, Grüneberg et al. (2019) compared soil C stocks down to a depth of 90 cm using data from the German national soil inventory. Over a period of ~25 years, C stocks in deep soils remained relatively stable overall. However, the study highlighted that soil type and site‐specific conditions play a crucial role in determining whether soils experience C losses or gains. A better understanding of deep‐soil C dynamics was identified as a critical research need, particularly with regard to its integration into Earth System Models (Hicks Pries et al. 2023).

In this study, we analyzed soil inventory data collected in 1984, 2012, and 2022 from 62 temperate European beech ( *Fagus sylvatica* ) stands to investigate C dynamics in mineral soils from 0 to 90 cm depth over almost four decades. These data were linked to dendrochronological records, soil chemical and physical properties, and information on stand and site characteristics. The forest stands span a gradient of ecosystem productivity and fertility, providing a robust basis for investigating the environmental factors predicting temporal dynamics in soil C. We address 3 specific research questions: (1) Have soils gained or lost C since the 1980s; and is there an effect of soil depth? (2) Which environmental factors predict changes in soil C? (3) How relevant are potential changes in deep soil C compared to C accumulation in tree biomass?

## 2 Materials and Methods

### 2.1 Study Area

**FIGURE 1:**
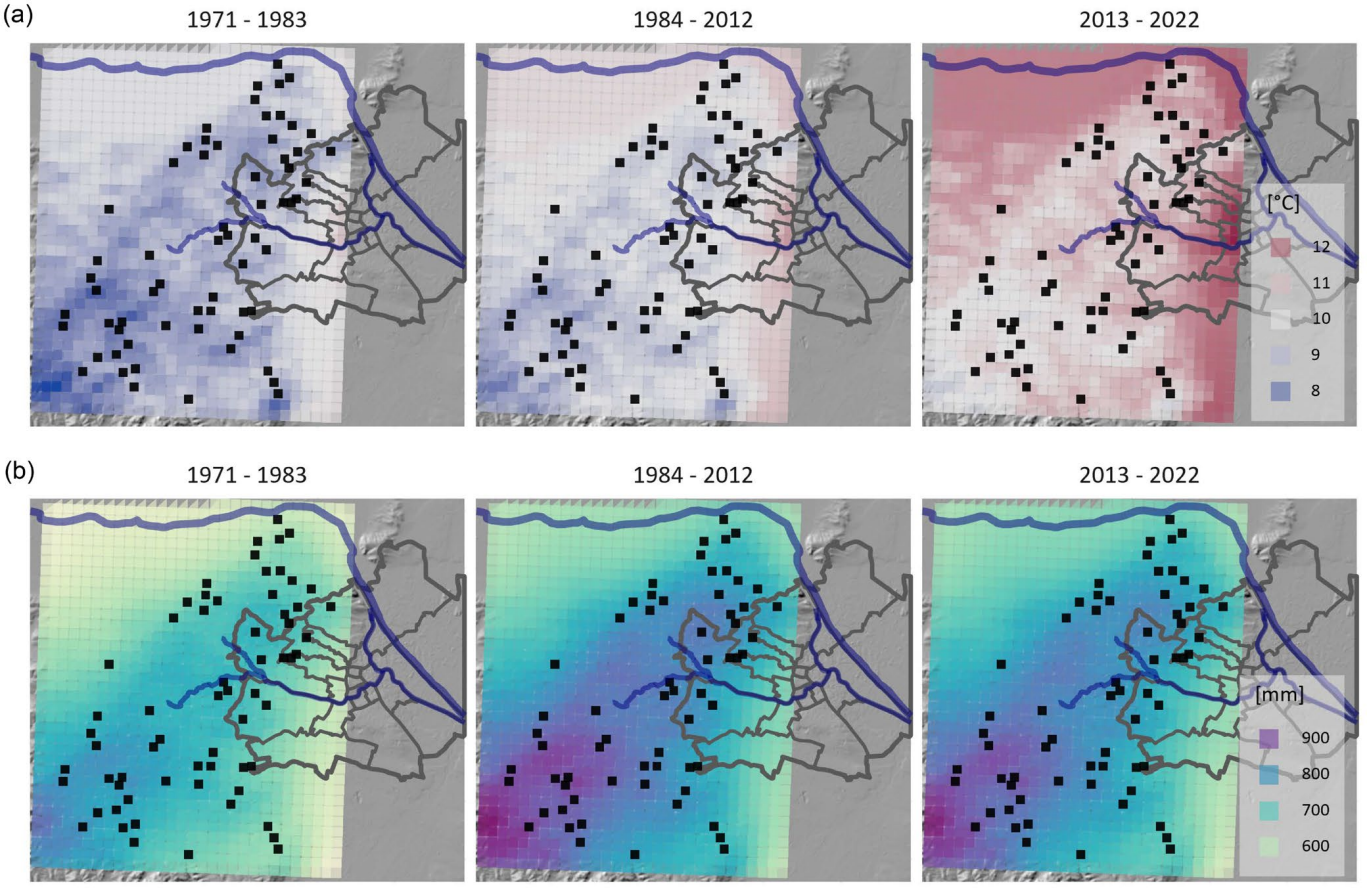
Mean annual air temperature (a) and precipitation (b) before and between the sampling years 1984, 2012, and 2022—in the Vienna Woods, Austria, with the 62 sampled forest stands depicted as black squares. The grey line indicates the border of the City of Vienna. Data are derived from Geosphere Spartacus (Hiebl and Frei 2016).

The study area is located in the Vienna Woods (Wienerwald), a forest landscape in close proximity to the City of Vienna, Austria (Figure 1), part of the UNESCO Biosphere Reserve “Wienerwald.” The predominant bedrock is Flysch, which consists mainly of tertiary and mesozoic sandstones and clay‐rich marls, while smaller areas in the southern part of the region are underlain by calcareous bedrock from the Northern Calcareous Alps (e.g., limestone and dolomite). The dominant soil types are Stagnosols and Cambisols, and the main humus form is Mull (IUSS Working Group WRB 2022). The total area spans approximately 125,000 ha, primarily consisting of forested land. Elevations in the region range from around 180 to over 800 m above sea level (a.s.l.). The mean annual precipitation varies between 600 and 900 mm, and the mean annual temperature ranges from 8°C to 9°C. The dominant tree species in the Vienna Woods is European beech ( *Fagus sylvatica* ). In 1984, 152 pure European beech stands were selected for sampling. Repeated sampling was conducted in 2012 and 2022, during which 97 and 62 of the original 152 beech stands were still available, as the remainder had been harvested (Figure 1). By 2022, the average stand age was 152 years, ranging from 108 to 238 years. The stands were not planted; natural regeneration has historically been the prevailing regeneration method and continues to be so. The resampled stands have not undergone any active management interventions since their initial selection in 1984. Further details on the stands and the study area can be found in Berger et al. (2016) and Table S1.

### 2.2 Soil Sampling

Soil sampling was conducted during the summers of 1984, 2012, and 2022, following the same methodology across all years. Seven soil cores (diameter: 5 cm, height: 5 cm) were collected from the 0‐ to 5‐cm mineral soil depth after carefully removing the organic layer. For deeper sampling, a soil auger (diameter: 2 cm, height: 100 cm) was used to collect samples from 30–40 and 80–90 cm mineral soil depths. Four replicates were taken per stand and pooled by depth for chemical analyses. Sampling was conducted within an area of approximately 0.1 ha per stand, with sampling locations spaced several meters apart to ensure representativeness. In cases where topsoil compaction occurred during coring, the compressed section was gently pushed back to its original position within the soil auger to ensure accurate sampling depth. This consistent sampling strategy allowed for direct comparisons across sampling years. In addition, during the 2022 campaign, two larger soil cores (diameter: 7 cm, length: 50 cm) were collected per stand for bulk density measurements and the determination of rock content and soil texture. Further details on the soil sampling procedure are available in Berger et al. (2016).

### 2.3 Soil Analyses

In the laboratory, soil samples were sieved at 2 mm prior to further analysis. In 1984, total C content was determined using a Wösthoff Carmhomat ADG 8 analyzer (Germany) according to ÖNORM L1080 and total S using LECO SC 132 analyzer (USA), while total N content was measured using the Kjeldahl method (2300 Kjeltec Analyzer Unit, Tecator, Sweden) following ÖNORM L1082. For the 2012 and 2022 samples, total C and N and S content (mg g^−1^) were analyzed using a LECO SC 444 analyzer (USA) in accordance with ÖNORM L1080. The methods were compared over several years, confirming that measurements were consistent and comparable across sampling periods. In 2012 and 2022, inorganic C was determined using the Scheibler method (ÖNORM L1084). No measurements of inorganic C were available for the 1984 samples. However, statistical analysis showed no significant differences in inorganic C between the 2012 and 2022 samples, while total and organic (total minus inorganic C) C content changed significantly (Figure S1), suggesting that inorganic C remained stable over time. Therefore, we attribute all observed changes in soil total C between the sampling years to the organic C fraction. Although we did not have archived intercalibration data from the 1980s, the consistent changes in organic C observed between 2012 and 2022—when identical instruments and protocols were used—support the robustness of the long‐term trends.

Exchangeable calcium (Ca), magnesium (Mg), and potassium (K) were extracted with 1 M ammonium acetate at pH 7 (ÖNORM L1086). Cations were analyzed by graphite furnace atomic absorption spectrometry (GF‐AAS, Perkin Elmer 3030, USA) in 1984 and by inductively coupled plasma optical emission spectrometry (ICP‐OES, Optima 3000 XL, Perkin Elmer, USA) in 2012 and 2022. Soil pH was measured using a glass Ag/AgCl combination electrode with a KCl reference electrode. For this, 10 g of soil was mixed with 25 mL of 0.1 M KCl, stirred, and allowed to settle overnight. The pH was measured 30 min after stirring the mixture again the next morning, following ÖNORM L1083. Further details on soil analyses are available in Berger et al. (2016).

The total iron (Fe) and manganese (Mn) content was determined through acid digestion, using nitric acid (65% HNO_3_) in 1984 and aqua regia (HCl/HNO_3_; ÖNORM EN 16174) in 2012 and 2022. The Fe and Mn concentrations were analyzed by graphite furnace atomic absorption spectrometry (GF‐AAS, Perkin Elmer 3030, USA) in 1984, and by inductively coupled plasma optical emission spectrometry (ICP‐OES, Optima 3000 XL, Perkin Elmer, USA) in 2012 and 2022. Both analytical methods were run in parallel for several years, yielding comparable results. Additional details on soil analyses are provided in Türtscher et al. (2017).

Root mass was determined for the 0–5 cm soil depth in 2012 and 2022. Roots were separated from soil, washed, dried at 105°C for 48 h, and subsequently weighed to calculate their mass per unit area (g m^−2^).

For 63 samples, sand, silt, and clay content was determined using wet sieving and particle size analysis (Sedigraph III, Micromeritics, GA, USA). For all other samples, soil texture was assessed by finger probing (ÖNORM L 1050). Sand, silt, and clay proportions from the measured samples were assigned to each texture class and transferred accordingly. Fine soil mass and its bulk density were determined in 2022 for the 0–5, 5–10, 10–20, 20–30, 30–40, and 40–50 cm soil depth intervals using samples collected with the 7‐cm soil corer. The soil was sieved through a 2‐mm mesh, and roots and stones were carefully removed and washed. Fine soil, roots, and stones were then dried at 105°C for 48 h and subsequently weighed to calculate their mass per unit area (g m^−2^) and bulk density (g cm^−3^).

Soil C and nutrient stocks were estimated for each stand and year of sampling. Soil C and nutrient concentrations were interpolated for depths between 0 and 5 cm, 30 and 40 cm, and 80 and 90 cm, respectively. Fine soil bulk density between the 50‐ and 90‐cm depth was assumed to be equivalent to that measured for the 40–50 cm depth interval. Soil C and nutrient stocks were calculated by multiplying soil element concentration by fine soil bulk density and correcting for rock content and soil penetration depth (estimated by means of soil auger). Fine soil bulk density was assumed to remain constant between 1984 and 2022. Inorganic C stocks were calculated for 2012 and 2022, and an average was calculated for each site assuming no change over time (see above and Figure S1). Subsequently, inorganic C stocks were subtracted from total soil C stocks of each year to obtain organic soil C stocks. For each stand, soil organic C stocks were calculated separately for three depth pools: topsoil (0–20 cm), midsoil (20–50 cm), and subsoil (50–90 cm). All stocks are expressed in Mg ha^−1^.

In addition to the depth‐based approach, we also applied an equivalent soil mass method to account for potential variation in bulk density across stands (Wendt and Hauser 2013). Soil organic carbon stocks were calculated for three equivalent soil mass layers (0–1000, 1000–2000, and 2000–3000 Mg fine soil ha^−1^), using a common cutoff of 3000 Mg ha^−1^ based on the stand with the lowest fine soil mass.

### 2.4 Dendrochronological Measurements

Wood core samples were collected from seven beech trees per study site in late 2022. Two cores were taken from opposite sides of each sampled tree to exclude reaction wood. The cores were examined using dendrochronological methods by cutting the cross section and digitizing each sample. The images of the cores were then measured using the WinDendro tree increment measuring software (Regent Instruments Inc., Quebec, Canada). Rinntech TSAP‐Win (Rinn 2003) was used to ensure the correct dating of each annual ring by cross‐dating the measurements. Thereafter, the dated tree rings were transferred into COFECHA (Holmes 1983) to assess the quality of the measurements. Core samples that could not be synchronized were omitted from the study.

To assess growth dynamics, the relationship between tree‐ring width and tree age was established. Subsequently, residuals between the age‐dependent growth trend and the observed ring widths were calculated. These residuals are interpreted as indicators of local growth conditions, where annual values greater than one represent above‐average growth, and values below one indicate reduced growth relative to the expected age‐related trend. Further details on dendrochronological measurements are available in Winter Artusio et al. (2025).

### 2.5 Stand and Site Characteristics

In the summer of 2022, the number of trees per stand and the diameter at breast height (DBH) were determined using angle‐count sampling (Avery and Burkhart 2015). The DBH for 1984 was estimated for the same trees by calculating diameter increments based on dendrochronological measurements. Above‐ground woody biomass per stand was then calculated for 1984 and 2022 using allometric functions (Forrester et al. 2017). The C content of the above‐ground woody biomass was estimated as 50% of the dry weight (de Wit et al. 2006) for each stand, and changes in C stocks between 1984 and 2022 were calculated accordingly.

In late summer 1984, 2012, and 2022, leaf samples of beech were collected from the upper crown of three trees per site. All subsamples per site were pooled before analysis. Total contents of C, N, and S were analyzed as described for the soil samples above. Phosphorus, Ca, Mg, and K were measured as total contents after digestion with HNO3/HClO4 (ÖNORM L1085) by GF‐AAS (1984 samples) and by ICP‐OES (2012 and 2022 samples), respectively. Further details on leaf analyses are available in Berger et al. (2016).

The vitality of mature trees was assessed based on crown dieback condition, using three classes: vital, minor stress, and major stress. The exposition and slope of each stand were measured by means of a compass and an inclinometer. Solar exposure was subsequently calculated based on exposition and slope. The elevation was taken from a terrain model.

Gridded meteorological data from the SPARTACUS dataset (Hiebl and Frei 2016) were obtained from the Austrian Meteorological Agency (GeoSphere Austria). Each forest stand was matched to its corresponding 1 × 1 km grid cell. The dataset provides daily records of mean temperature and precipitation from 1961 to the present. These data were used to calculate mean annual temperature and total annual precipitation for the specified time periods.

Gridded N and S deposition data (wet and dry) were obtained from the EMEP MSC‐W model output as reported in the EMEP Status Report 1/2023 (Fagerli et al. 2023). The data have a spatial resolution of 0.1° (~10 × 10 km), and each forest stand was assigned to the corresponding grid cell based on its geographic coordinates. Deposition values were extracted for the years 1990 (used as a proxy for pre‐1990 conditions), 2012, and 2022, corresponding to the soil sampling years. In addition, average deposition across these years was calculated. Changes in N and S deposition between 1990 and 2022 were quantified, with S changes serving as a proxy for declining acid rain exposure.

### 2.6 Statistical Analysis

Prior to analysis, changes in soil C and nutrient contents and organic C stocks were calculated as the difference between the sampling years 2012 and 2022 and the baseline year 1984. Linear mixed‐effects models were used to test whether changes in soil C and nutrients and organic C stocks differed significantly from zero, with stands included as random effects. For content data, each soil depth was analyzed separately.

A linear mixed effects model with stand as a random effect was used to compare root mass between sampling years 2012 and 2022. A non‐parametric Wilcox test was used to compare tree ring increment percentage between growth periods 1950–1984 and 1984–2022.

To investigate the influence of environmental factors on changes in soil C stocks between 1984 and 2022, available environmental variables were reduced to 10 composite variables using principal component analysis (PCA) to simplify analysis and address potential multicollinearity among variables (Figure S2). Separate PCAs were performed for soil chemistry, soil physical properties, leaf nutrients, stand properties, and climate variables. The first and second PCA axes from each analysis were used as composite environmental variables representing *soil nutrients and metals, soil calcareousness, soil clay content, soil stone content, leaf CaMg content, leaf NPKS content, stand productivity, stand age, local exposure*, and *regional aridity* of each stand (Figure S2).

Structural equation modelling was subsequently used to test for relationships between composite environmental variables on soil C stock changes and among them (Beaujean 2014). Nitrogen and S deposition data, including absolute values and changes between 1990 and 2022, were included as additional variables in the model. Potential collinearity between composite variables derived from different PCAs was accounted for within the structural equation modelling framework, which is particularly well‐suited for modeling complex relationships among correlated predictors. To obtain the best‐fitting model, pathways and variables were removed in a stepwise procedure; significant relations (*p* < 0.05) were subsequently retained. All statistics were conducted in R (R Core Team 2024) using the “nlm” package (Pinheiro et al. 2017) for linear mixed effects models, the “lavaa” package for structural equation modelling (Rosseel 2012) and “ggplot” for graphics (Wickham 2016). Level of significance for all statistical analyses was set at *p* < 0.05.

## 3 Results

### 3.1 Changes in Carbon Content and Stocks

**FIGURE 2:**
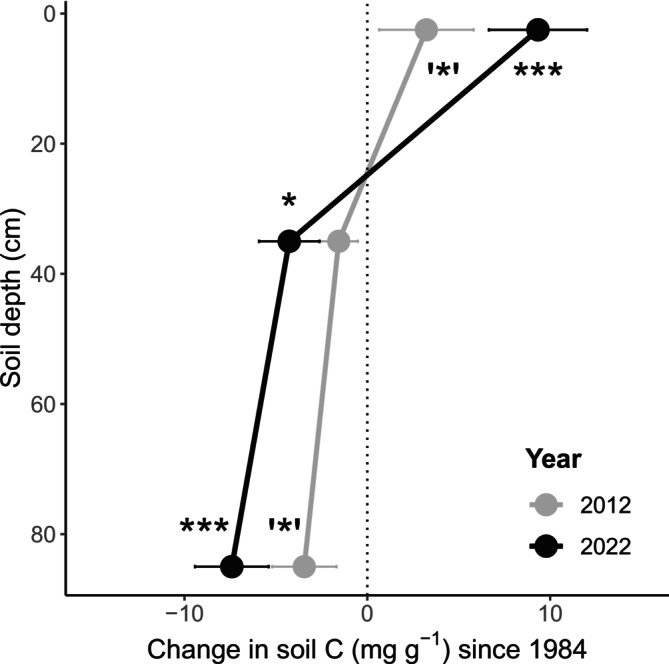
Changes in soil carbon (C) content at three depths of European beech stands in the Vienna Woods, Austria, in 2012 and 2022 as compared to 1984 (mean ± SE; *n* = 62). Significant differences from zero are indicated by asterisks (***p < 0.001; *p < 0.05; ‘*’*p* < 0.1).

Across the 62 European beech stands, changes in total soil C content between 2012 and 2022, compared to 1984, showed distinct depth‐dependent patterns (Figure 2). At 0–5 cm depth, C content increased by 3.2 ± 2.6 mg g^−1^ (mean ± SE, here and throughout text) in 2012 and by 9.34 ± 2.7 mg g^−1^ in 2022, starting from an initial content of 58.8 ± 4.3 mg g^−1^ in 1984. At 30–40 cm depth, C content showed no significant difference in 2012 but decreased by −4.27 ± 1.7 mg g^−1^ in 2022, from an initial value of 22.5 ± 3.5 mg g^−1^ in 1984. At 80–90 cm depth, C content declined by −3.4 ± 1.8 mg g^−1^ in 2012 and by −7.4 ± 2.0 mg g^−1^ in 2022, starting from 21.6 ± 4.3 mg g^−1^ in 1984. Since inorganic C content remained stable over time (Figure S1), we attribute these changes to the organic C fraction.

**FIGURE 3:**
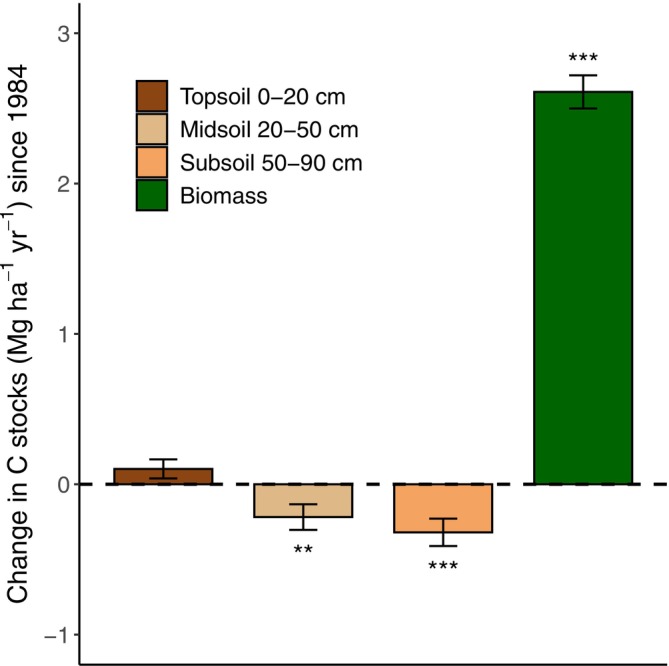
Changes in organic carbon (C) stocks in soil and aboveground biomass of European beech stands in the Vienna Woods, Austria (mean ± SE; *n* = 62), between the sampling campaigns in 1984 and 2022. Significant differences from zero are indicated by asterisks (***p < 0.001; **p < 0.01).

Average organic C stocks in mineral soil in 1984, 2012, and 2022 were estimated at 59.9 ± 4.2, 61.6 ± 3.8, and 63.8 ± 3.6 Mg ha^−1^ in the topsoil (0–20 cm), 48.8 ± 4.5, 46.4 ± 3.6, and 40.5 ± 3.2 Mg ha^−1^ in the midsoil (20–50 cm), and 31.0 ± 4.0, 27.7 ± 3.1, and 18.8 ± 1.8 Mg ha^−1^ in the subsoil (50–90 cm), respectively (Figure S3). Between 1984 and 2022, topsoil organic C stocks increased by 0.10 ± 0.06 Mg C ha^−1^ year^−1^, while midsoil and subsoil stocks decreased by −0.22 ± 0.09 and −0.32 ± 0.09 Mg C ha^−1^ year^−1^, respectively (Figure 3). However, only changes in mid‐ and subsoil stocks were statistically significant (*p* < 0.05). Overall, soils lost −0.44 ± 0.19 Mg C ha^−1^ year^−1^ between 1984 and 2022.

Results from the equivalent soil mass approach confirmed the same overall pattern: organic C stocks increased in the 0–1000 Mg ha^−1^ fine soil layer (topsoil), slightly declined in the 1000–2000 Mg ha^−1^ layer (midsoil; not significant), and significantly declined in the 2000–3000 Mg ha^−1^ layer (subsoil). However, changes were slightly smaller in magnitude than those based on fixed depth; due to the constrained soil mass cutoff (Figure S4).

Aboveground stand biomass was 131.4 ± 5.5, 208.6 ± 7.3, and 230.5 ± 7.7 Mg C ha^−1^ in 1984, 2012, and 2022, respectively (Figure S3). On average, aboveground stand biomass increased by 2.8 ± 0.12, 2.2 ± 0.10, and 2.6 ± 0.11 Mg C ha^−1^ year^−1^ between 1984 and 2012, 2012 and 2022, and 1984 and 2022, respectively (Figure 3).

Root biomass in 0–5 cm soil depth was 3.21 ± 0.36 and 4.14 ± 0.36 Mg ha^−1^ in 2012 and 2022, respectively (Figure S5) and the differences between the years were statistically significant.

### 3.2 Environmental Factors Determining Soil Carbon Change

**FIGURE 4:**
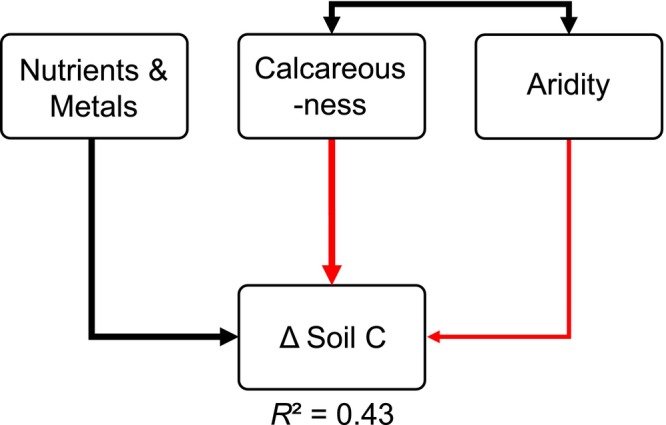
Structural equation model illustrating the influence of composite environmental variables on changes in soil organic carbon (C) stocks between the sampling years 1984 and 2022 in European beech stands of the Vienna Woods, Austria. Composite environmental variables are derived from the first and second axes of principal component analyses of soil, site, and climate variables (see Figure S2). Single‐headed arrows indicate significant relationships, while double‐headed arrows represent significant correlations between variables. Black arrows indicate positive relationships or correlations, whereas red arrows indicate negative ones. The thickness of the arrows reflects the strength of the respective paths. Soil C loss is signified by negative numbers of *Δ* Soil C. A negative relationship between *Δ* Soil C and a potential driver (e.g., aridity) therefore means that soil C losses are stimulated at greater aridity.

The developed structural equation model explained 43% of the variation in changes in soil organic C stocks in the study region (Figure 4). A non‐significant chi‐squared test (*p* > 0.05) indicated a good fit between the model and the data (Grace 2006). The model revealed a direct positive effect of the composite variable *soil nutrients and metals*, and direct negative effects of *soil calcareousness* and *regional aridity. Soil nutrients and metals* was defined by soil total Fe, total Mn, and exchangeable K stocks, as well as the soil C to N ratio (Figure S2). Stands with high nutrient and metal stocks and low C to N ratios experienced less soil C loss compared to those with low nutrient and metal stocks and high C to N ratios. *Soil calcareousness* was determined by soil exchangeable Ca, exchangeable Mg, and inorganic C stocks, along with soil pH. Stands with calcareous soils showed greater soil C loss than those with less calcareous influence. These calcareous soils also had higher soil organic C and N stocks. *Regional aridity* was characterized by elevation, mean annual precipitation, and mean annual temperature. Stands at lower elevations with drier, warmer conditions experienced greater soil C loss than stands at higher elevations with cooler, moister conditions. Finally, a positive correlation between *regional aridity* and *soil calcareousness* indicates that warmer, drier stands tend to have more calcareous soils which were more present in the south of the study area. No significant relationship was found between *nutrients and metals* and *soil calcareousness*, or between *nutrients and metals*, and *regional aridity*. Other environmental variables including *soil clay content, soil stone content, leaf CaMg content, leaf NPKS content, stand productivity, stand age, local exposure*, as well as *atmospheric deposition fluxes* or *changes in deposition fluxes* of reactive N and S from gridded data did not improve the structural equation model explaining soil C change and were therefore not retained.

## 4 Discussion

Predicting the response of deep‐soil C to global change remains a critical research priority, yet long‐term field observations for forest ecosystems are notably scarce (Schrumpf et al. 2011; Hicks Pries et al. 2023). Here, we present evidence of significant deep‐soil C losses in temperate European beech ( *Fagus sylvatica* ) forests since the 1980s, which outweighed topsoil C gains and decreased the ecosystem's C sink capacity.

### 4.1 Deep‐Soil Carbon Losses Outweigh Topsoil Gains

We relate the increase in topsoil C primarily to increased litter inputs. This may result from the natural accumulation of tree biomass over time, contributing to higher litter production from both leaf and root biomass (Wutzler et al. 2008; Forrester et al. 2017). Rising temperatures and increased precipitation since the 1980s (Figure 1), combined with a fertilization effect from elevated atmospheric CO_2_ concentrations and still relatively high N deposition rates—despite a decline over recent decades (Table S1)—may have further accelerated tree growth and litter inputs (Liu et al. 2005; Pretzsch et al. 2014; Kim et al. 2020; Gao et al. 2023). In support of this, our dendrochronological analysis revealed a 7% higher tree ring increment (corrected for tree age) between 1984 and 2022 compared to 1950–1984 (Figure S6), indicating a substantial increase in tree growth. Topsoil root biomass also increased by approximately 1 Mg ha^−1^ between 2012 and 2022 (Figure S5), suggesting greater inputs of root litter and exudates to soil (Brunner et al. 2013; Chari et al. 2024). Increased precipitation and enhanced water availability in the topsoil may have also stimulated root growth or even facilitated an upward shift in the rooting zone (Guswa 2008; Fan et al. 2017; Zou et al. 2022; Jaeger et al. 2024). The role of soil nutrients as drivers of these changes remains uncertain. While N and Ca concentrations increased, K initially decreased in 2012 before recovering in 2022 (Figure S7). Shifts in nutrient availability may have influenced both tree root growth and litter decomposition, and thus C dynamics. In contrast, a decline in leaf nutrient concentrations—except for N and S—since the 1980s suggests a decline in litter quality (Figure S8). Lower concentrations of key nutrients such as P, K, or Ca in leaf litter may have reduced its decomposability (Zhang et al. 2008; Prescott and Vesterdal 2021). Since a synthesis of stable isotope studies showed that litter C transfer to soil is higher for slow decomposing litter than for fast decomposing litter, it is plausible that this mechanism has contributed to the observed increase in soil C (Zheng et al. 2021).

The observed deep‐soil C loss could be related to a warming‐induced acceleration of microbial decomposition, likely driven by the ~2°C temperature increase since the 1980s (Hicks Pries et al. 2017; Nottingham et al. 2020; Soong et al. 2021; Wang et al. 2022). This assumption is supported by global model simulations showing that, in temperate regions, soil temperature increases at 1‐m depth closely track air temperature changes, with only minor lags in warming (Soong et al. 2020). The larger temperature increases between 2012 and 2022, compared to the increase between 1984 and 2012 (Figures 1 and S9), may explain why soil C changes occurred in a similar range despite the shorter interval between sampling in the second study period. Moreover, in deeper horizons, it is likely that water availability during summer drought phases stayed higher and combined with warmer temperatures may have sustained microbial activity, accelerating C losses from the subsoil. Increased temperatures in the topsoil may also have raised concentrations of dissolved organic C, which, as proposed by Soong et al. (2021), could be transported downward into deeper horizons. This effect may have been further enhanced by increased surface litter inputs, which likely contributed to higher dissolved organic C production and vertical leaching into deeper soil layers. This influx of labile C may alleviate C limitations for microbial communities in the subsoil, stimulating the decomposition of deep soil C (Fontaine et al. 2007). Rhizosphere priming could also have contributed to C losses at depth, particularly if enhanced tree growth and nutrient demand led to higher root activity in deeper soil horizons under changing environmental conditions (Jackson et al. 2019). Additionally, temperature sensitivity has been shown to increase with soil depth, potentially accelerating deep‐soil C losses more strongly than topsoil C losses with warming (Karhu et al. 2010; Yan et al. 2017). Further, microbial communities in subsoils can differ in composition and function from those in the topsoil (Frey et al. 2021). Changes in soil nutrients may also be linked to the observed C decline, though interpretation remains complex (Figure S7). A stable N content and the resulting decrease in the soil C to N ratio may have enhanced organic matter decomposition (Xu et al. 2016), while increased Ca could have counteracted this effect through organic matter stabilization by organic‐Ca complexes (Rowley et al. 2018). The role of elevated K also remains unclear, but it has been shown that fertilization with N and K can stimulate soil organic matter decomposition (Soong et al. 2018), potentially increasing C loss. In addition to climate warming, reductions in atmospheric deposition may also have contributed to the observed losses of deep‐soil C. Specifically, declines in N deposition and acid rain across Europe since the 1990s may have altered soil chemical conditions and microbial activity. Lower N inputs can shift microbial nutrient limitations and potentially increase soil organic matter mineralization (Janssens et al. 2010; Liu and Greaver 2010). Similarly, declining acid deposition can raise soil pH, potentially enhancing microbial activity and decomposition rates (Evans et al. 2008). While our structural equation model did not identify N or S deposition changes as significant predictors of soil C change, the long‐term shifts in deposition remain important background drivers and may have interacted with other factors in complex ways.

The depth‐dependent pattern of soil C changes observed across 62 forest stands in our study is consistent with previous research in other regions. For instance, in a whole‐soil warming experiment conducted in a mixed coniferous forest, soil C stocks in horizons between 0 and 20 cm increased after approximately 5 years of 4°C warming treatment, while C stocks in horizons between 30 and 90 cm decreased by −32.1 Mg C ha^−1^, equivalent to a 33% reduction (Soong et al. 2021). Similarly, a study from Germany comparing data from the national forest soil inventories reported a significant increase in C content at 0–5 cm soil depth, mixed increases at 5–10 cm soil depth, and an almost exclusive decrease at 10–30 cm soil depth (Grüneberg et al. 2014). A subsequent re‐analysis of the same data, nevertheless, reported an overall increase of 0.69 Mg ha^−1^ year^−1^ in C stocks for the 0–30 cm horizon, while no changes were detected below 30 cm (Grüneberg et al. 2019). However, these differences—compared to our results—may be explained by geographic variation: sandy soils in northeastern Germany contributed to the observed increases in C, whereas soils in mountainous and hilly regions in southern Germany—characterized by substrates such as weathered limestone, marl, or clay, like those in the Vienna Woods—showed decreases in soil C of up to −0.7 Mg ha^−1^ year^−1^. This is consistent with our findings. Further, a study from France investigating data from inventories in 1994 and 2008 showed an increase in C stocks at 0–10 cm soil depth and a slight, though not statistically significant, decrease at 30–40 cm soil depth (Jonard et al. 2017). However, this study did not examine soil horizons deeper in the profile, leading to their conclusion that forest soils sequestered an average of +0.35 Mg C ha^−1^ year^−1^. This underscores the need to include deep‐soil horizons to accurately assess the overall soil C balance.

### 4.2 Environmental Factors Determining Soil Carbon Change

We identified forest stands with soils rich in total Fe and Mn to be less prone to C loss. Iron oxides play a crucial role in stabilizing soil C via mineral‐organic matter associations (Gu et al. 1994; Lehmann and Kleber 2015b; Jia et al. 2024). Iron oxides can adsorb dissolved and other forms of organic C, forming stable Fe‐organic matter complexes. This process is particularly effective in soils with high Fe content, where Fe‐organic matter complexes protect organic C from microbial mineralization (Sodano et al. 2017; Jeewani, Ling, et al. 2021; Jeewani, Van Zwieten, et al. 2021). The formation of Fe‐organic matter complexes is influenced by the content and type of Fe oxides and the C to Fe molar ratio, with higher Fe concentrations leading to more significant stabilization of organic C (Jeewani, Ling, et al. 2021; Jeewani, Van Zwieten, et al. 2021). Iron's redox properties allow it to interact dynamically with soil organic C, especially in soils where periodic flooding occurs. The reduction and oxidation of Fe can lead to the formation of fresh Fe surfaces that bind organic C, enhancing its stabilization (Adhikari and Yang 2015; Hu et al. 2024). This could be an important mechanism underlying soil C stabilization at sites with temporally waterlogged Stagnosols on Flysch bedrock. If such waterlogged conditions persist and become reducing, however, Fe mobilization could impair organo‐mineral associations and reduce the soil's potential for C stabilization. In contrast, Mn can both destabilize and stabilize soil C; it has a high potential to degrade organic molecules through abiotic and microbially mediated oxidation and to stabilize organic molecules, at least temporarily, through organo‐mineral associations (Li et al. 2021).

Calcareous soils, in contrast, were identified as more vulnerable to C loss. Calcium can play a significant role in stabilizing soil C by promoting the formation of organo‐mineral associations through interbridging negative charges between clay minerals and organic matter (Rowley et al. 2018; Shabtai et al. 2023). This aligns with our observation that calcareous soils, which are rich in Ca (and Mg), contained higher organic C and N stocks (Figure S2). However, while high Ca concentrations may have contributed to soil C accumulation, these stocks were more vulnerable to environmental changes. Multiple studies have demonstrated that the extent of soil C loss in response to climate warming, CO_2_ fertilization, afforestation, and forest disturbances is often proportional to the initial size of the C stock (Prietzel et al. 2016; Hong et al. 2020; Terrer et al. 2021; Mayer et al. 2024). This suggests that while Ca‐rich soils may store substantial amounts of organic C, they may also be more susceptible to destabilization under shifting environmental conditions. Further, calcareous sites were characterized by a warmer and drier climate compared to other sites (Figures 4 and S2), suggesting that an increase in precipitation since the 1980s may have enhanced decomposition more strongly than in cooler, wetter regions. Our results are, nevertheless, in line with the aforementioned German inventory study by Grüneberg et al. (2019), who identified calcareous soils as particularly prone to C loss. These findings highlight the complex interplay of soil chemistry, climate, and environmental changes in shaping soil C dynamics.

### 4.3 Soil Carbon Losses Reduced Ecosystem Carbon Sink

From a climate perspective, the key question is whether the ecosystem's net CO_2_ sink capacity remains positive, which depends on the balance between C sequestration in plant biomass and C loss through C mineralization by microorganisms. Here, the estimated average stand biomass in 1984 was 131 Mg C ha^−1^, increasing by 2.6 Mg C ha^−1^ year^−1^ until 2022. These values are broadly comparable to other mature European beech stands (Bascietto et al. 2004; Joosten et al. 2004; Schulte‐Bisping et al. 2012; Nagel et al. 2023). However, when these biomass gains are compared to C losses from soil, the net CO_2_ sink capacity was reduced by 17%. When scaled to the Vienna Woods region, where approximately 80,000 ha are forested and 20% of this area consists of stands older than 100 years (ÖBf‐AG 2025), these soil C losses translate to an estimated total loss of roughly 1 Mt CO_2_ between 1984 and 2022. Notably, this outcome would differ if only C gains in the topsoil were considered, as the soil C balance turns negative only when subsoil losses are accounted for (Figure 3). This highlights the critical role of soil, and of deep‐soil in particular, in determining the long‐term carbon balance of forest ecosystems, even in regions with substantial biomass growth.

## 5 Conclusions

In conclusion, our study shows significant depth‐dependent changes in soil organic C stocks in temperate mature European beech forests since the 1980s, with large losses below 20 cm. These results highlight the importance of including deep‐soil C in forest ecosystem assessments, as it plays a key role in the overall C balance. Organic‐rich calcareous soils appear to be particularly vulnerable to C loss, probably because they are less effective at stabilizing C than Fe and Mn rich soils. While warming has likely driven much of the deep‐soil C loss by accelerating microbial decomposition, other factors such as changes in litter inputs or rooting depth may also have contributed to the observed patterns. Our results highlight the need for long‐term monitoring of deep‐soil C to better predict how forests will respond to global change and to improve C accounting in models.

## Author Contributions

**Mathias Mayer:** conceptualization, formal analysis, investigation, methodology, validation, writing – original draft. **Klaus Dolschak:** data curation, formal analysis, methodology, resources, visualization, writing – review and editing. **Emilia Winter Artusio:** data curation, methodology, validation, writing – review and editing. **Michael Grabner:** data curation, methodology, resources, investigation, writing – review and editing. **Michael Tatzber:** data curation, methodology, resources, writing – review and editing. **Iftekhar U. Ahmed:** data curation, investigation, methodology, writing – review and editing. **Elisabeth Wächter:** data curation, formal analysis, methodology, writing – review and editing. **Selina Türtscher:** data curation, investigation, methodology, writing – review and editing. **Leopold Lindebner:** data curation, methodology, writing – review and editing. **Isolde K. Berger:** investigation, methodology, writing – review and editing. **Pétra Berger:** investigation, methodology, writing – review and editing. **Wolfgang Wanek:** data curation, methodology, resources, investigation, writing – review and editing. **Torsten W. Berger:** conceptualization, formal analysis, funding acquisition, investigation, methodology, project administration, supervision, validation, writing – original draft.

## Conflicts of Interest

The authors declare no conflicts of interest.

## Supporting information


**Data S1:** gcb70446‐sup‐0001‐DataS1.pdf.

## Data Availability

The data that support the findings of this study are openly available in EDI Data Portal at https://doi.org/10.6073/pasta/fee64b0ef91afb656217209fba1519c3.
