# Supplementary material for: Substantial Deep‐Soil Carbon Losses Outweigh Topsoil Gains in European Beech Forests Since the 1980s
Source: Glob Chang Biol. 2025 Sep 1;31(9):e70446. doi: 10.1111/gcb.70446 (PMC12401508; doi:10.1111/gcb.70446)
Supplement: Supplementary file 1 — Data S1: gcb70446‐sup‐0001‐DataS1.pdf. [file GCB-31-e70446-s001.pdf]

Supporting information

Substantial deep-soil carbon losses outweigh topsoil gains in European beech forests since the 1980s

Mathias Mayer[1,5*], Klaus Dolschak[1], Emilia Winter Artusio[1, 2], Michael Grabner[2], Michael Tatzber[3], Iftekhar U. Ahmed[1, 3], Elisabeth Wächter[2], Selina Türtscher[1], Leopold Lindebner[1], Isolde K. Berger[1], Pétra Berger[1, 4], Wolfgang Wanek[4], Torsten W. Berger[1]

[1]Institute of Forest Ecology, Department of Ecosystem Management, Climate and Biodiversity, BOKU University, Peter Jordan-Straße 82, 1190 Vienna, Austria

[2]Institute of Wood Technology and Renewable Materials, Department of Natural Sciences and Sustainable Resources, BOKU University, Konrad Lorenz-Straße 24, 3430 Tulln an der Donau, Austria

[3]Austrian Research Centre for Forests (BFW), Department of Forest Protection, Seckendorff-Gudent-Weg 8, 1131 Vienna, Austria

[4]Centre for Microbiology and Environmental Systems Science, Division of Terrestrial Ecosystem Research, University of Vienna, Djerassiplatz 1, 1030 Vienna, Austria

[5]Forest Soils and Biogeochemistry, Swiss Federal Institute for Forest, Snow and Landscape Research (WSL), Zürcherstrasse 111, 8903 Birmensdorf, Switzerland

*Corresponding author

TABLE S1 Summary statistics (mean and standard error, SE; n = 62) of soil chemical and physical properties, leaf nutrient concentrations, stand properties, site and climate variables, and atmospheric deposition for 62 European beech stands in the Vienna Woods, Austria. These data were used in principal component analyses (PCA) to derive composite environmental variables (see Fig. S2). Note: vitality and location classes are excluded, as they represent categorical codes whose numeric values are not meaningful without further classification details. The derived solar exposure index is also excluded, as its numeric values are not directly interpretable without additional context.
(Table continued on next page)

	Mean	SE	Unit
Soil chemistry (0-90 cm)			
Organic C	139.7	11.2	Mg ha^{-1}
Inorganic C	18.9	5.3	Mg ha^{-1}
Soil total N	8.4	0.6	Mg ha^{-1}
Soil C:N ratio	17.6	0.8	-
Soil exch. Ca	16.4	1.7	Mg ha^{-1}
Soil exch. Mg	1.3	0.1	Mg ha^{-1}
Soil exch. K	0.7	0	Mg ha^{-1}
Soil pH (KCl)	4.8	0.2	-
Soil total Mn	5.1	0.5	Mg ha^{-1}
Soil total Fe	166.2	9.5	Mg ha^{-1}
Soil physics			
Clay	31	1.4	%
Sand	34	1.7	%
Silt	35	0.8	%
Soil penetration depth	81.3	1.9	cm
Coarse soil	1223	131	Mg ha^{-1}
Fine soil	4508	162	Mg ha^{-1}
Slope	13	1	°
Leaf nutrients			
Leaf N	22.6	0.31	mg g^{-1}
Leaf P	1.1	0.03	mg g^{-1}
Leaf K	12.0	0.37	mg g^{-1}
Leaf Ca	12.1	0.46	mg g^{-1}
Leaf Mg	2.1	0.10	mg g^{-1}
Leaf S	1.5	0.02	mg g^{-1}
Stand properties			
Stand age	152	3	years
Number of trees	285	16	N ha^{-1}
Biomass C 1984	131.4	5.5	Mg ha^{-1}
Biomass C 2022	230.5	7.7	Mg ha^{-1}
Crown closure	80	2	%

TABLE S1 (continued) Summary statistics (mean and standard error, SE; n = 62) of soil chemical and physical properties, leaf nutrient concentrations, stand properties, site and climate variables, and atmospheric deposition for 62 European beech stands in the Vienna Woods, Austria. These data were used in principal component analyses (PCA) to derive composite environmental variables (see Fig. S2). Note: vitality and location classes are excluded, as they represent categorical codes whose numeric values are not meaningful without further classification details. The derived solar exposure index is also excluded, as its numeric values are not directly interpretable without additional context.
(Table continued on next page)

	Mean	SE	Unit
Climate			
MAP	799	6	mm yr^{-1}
MAT	10	0	°C
Elevation	407	10	m a.s.l.
Atmospheric deposition			
N deposition 1990	18.7	0.4	kg ha^{-1}
N deposition 2012	12.0	0.2	kg ha^{-1}
N deposition 2022	10.8	0.3	kg ha^{-1}
S deposition 1990	18.9	0.6	kg ha^{-1}
S deposition 2012	2.8	0.0	kg ha^{-1}
S deposition 2022	1.6	0.0	kg ha^{-1}
Change in N deposition	-7.9	0.1	kg ha^{-1}
Change in S deposition	-17.3	0.6	kg ha^{-1}

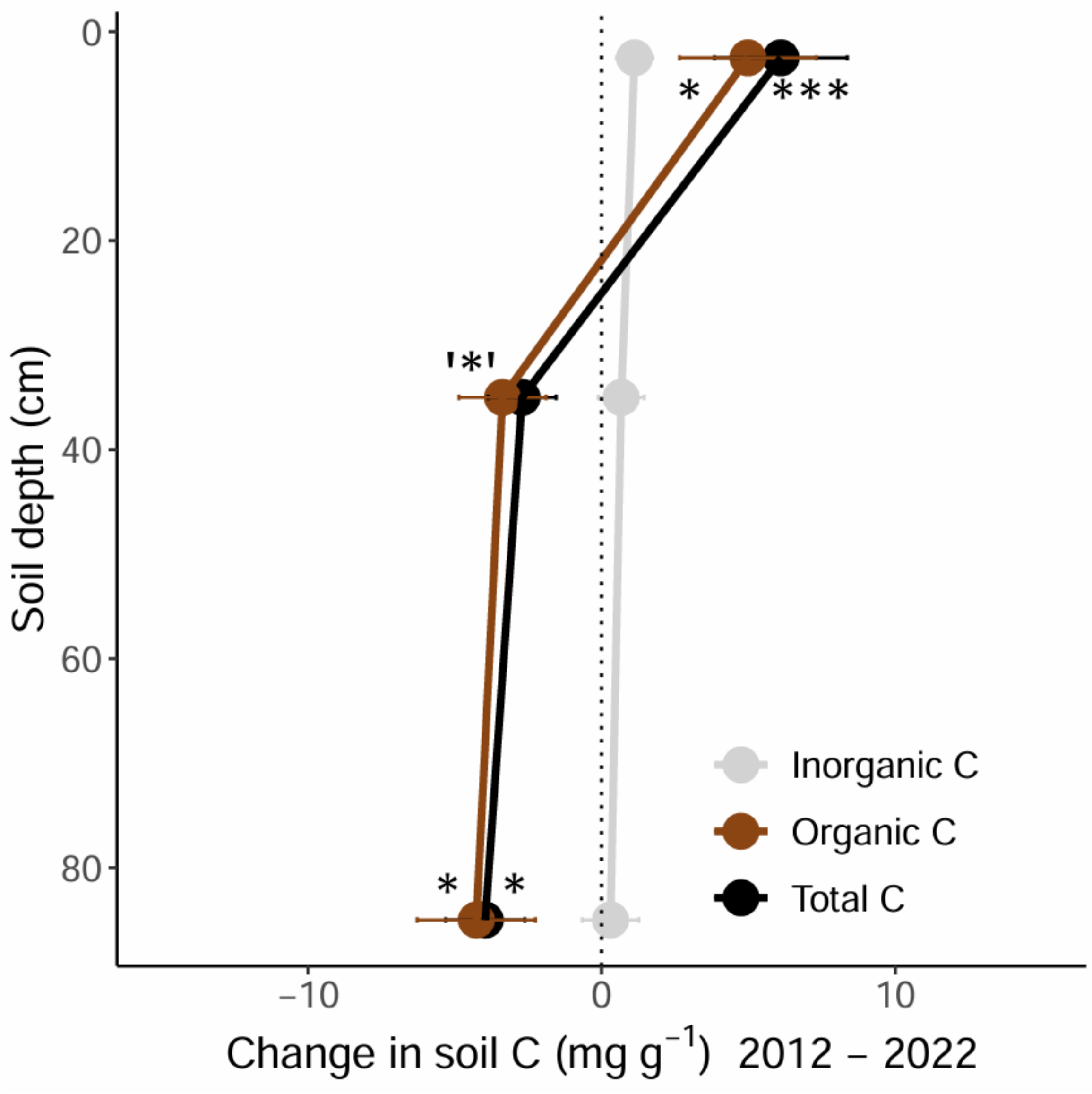

FIGURE S1 Changes in total, organic, and inorganic soil carbon (C) content at three depths of European beech stands in the Vienna Woods, Austria, in 2022 as compared to 2012 (mean ± SE; n = 62). Significant differences from zero are indicated by asterisks (***p < 0.001; *p < 0.05; '*'p < 0.1).

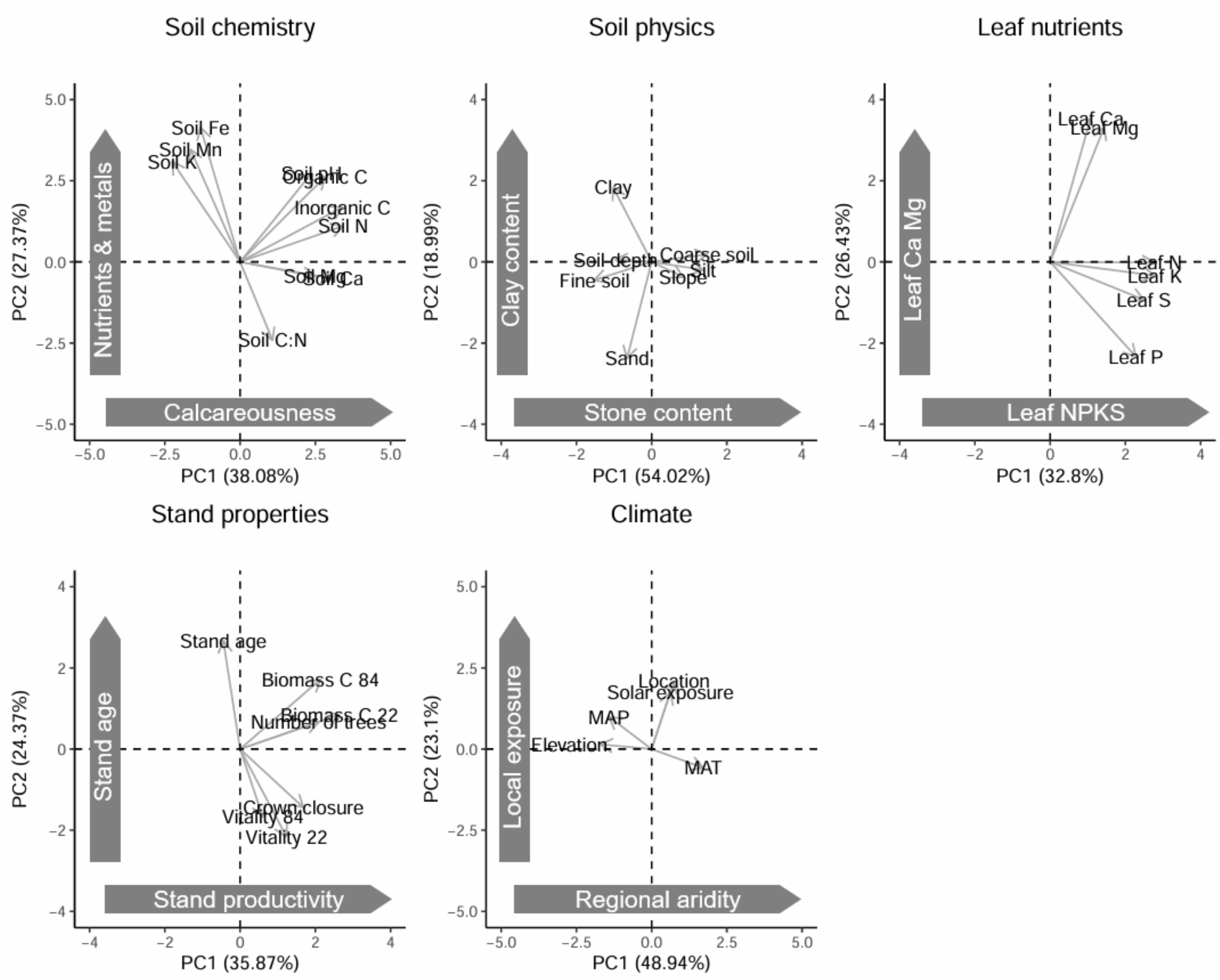

FIGURE S2 Principal component analyses (PCA) analysing soil chemical and physical properties, leaf nutrient concentrations, stand properties, as well as site and climate variables of 62 European beech stands in the Vienna Woods, Austria. The first and second axis of the PCA's were used as composite environmental variables in a structural equation model (Fig. 4).

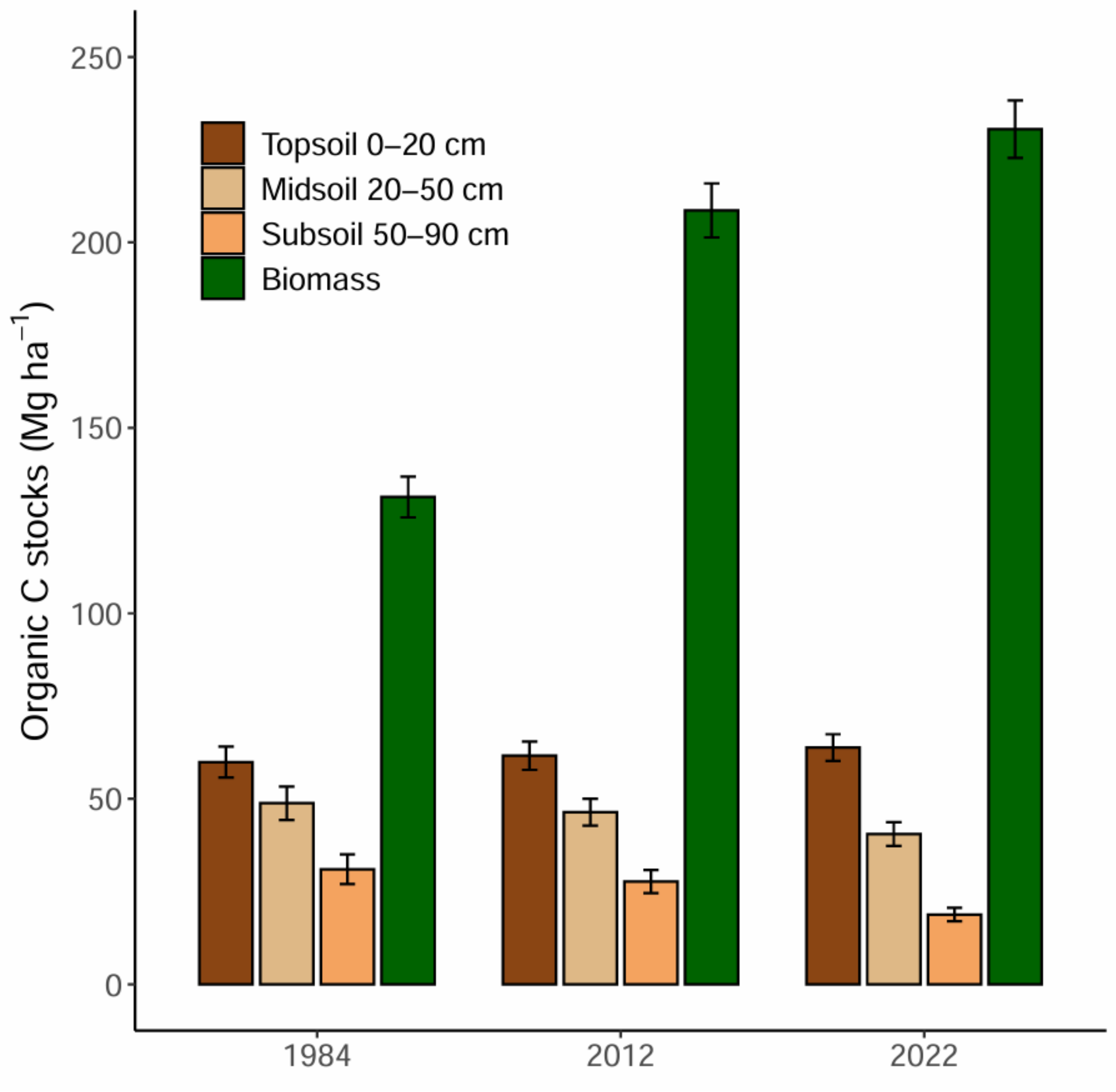

FIGURE S3 Soil organic carbon (C) stocks in different soil depths and aboveground stand biomass C stocks (mean ± SE; *n* = 62) of European beech stands in the Vienna Woods, Austria, for the sampling campaigns in 1984, 2012 and 2022.

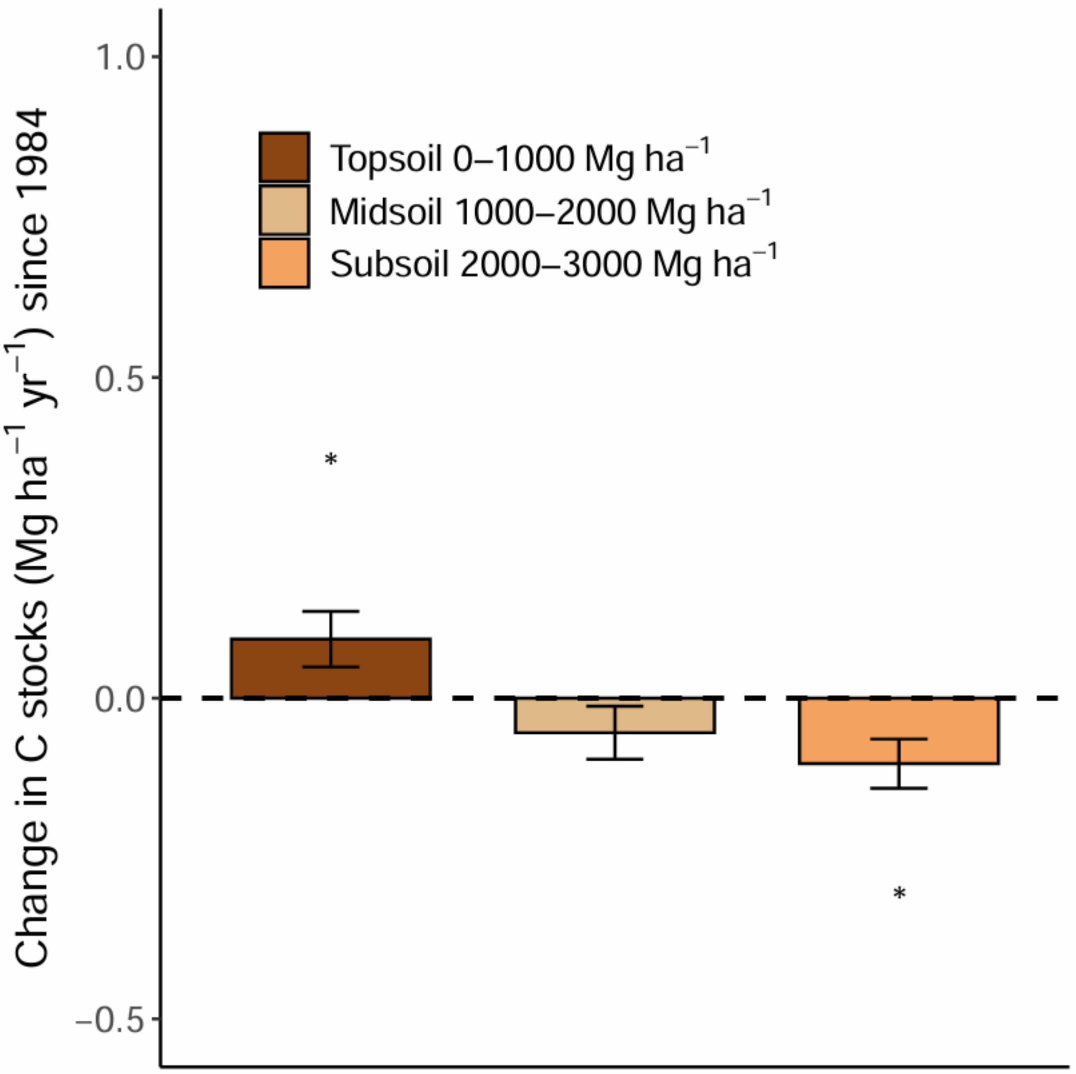

FIGURE S4 Changes in organic carbon (C) stocks in mineral soil of European beech stands in the Vienna Woods, Austria, between 1984 and 2022, calculated using the equivalent soil mass (ESM) approach (mean ± SE; n = 62). Soil layers represent cumulative fine soil masses of 0–100, 100–200, and 200–300 Mg ha^{-1}. Significant differences from zero are indicated by asterisks (* $p < 0.05$).

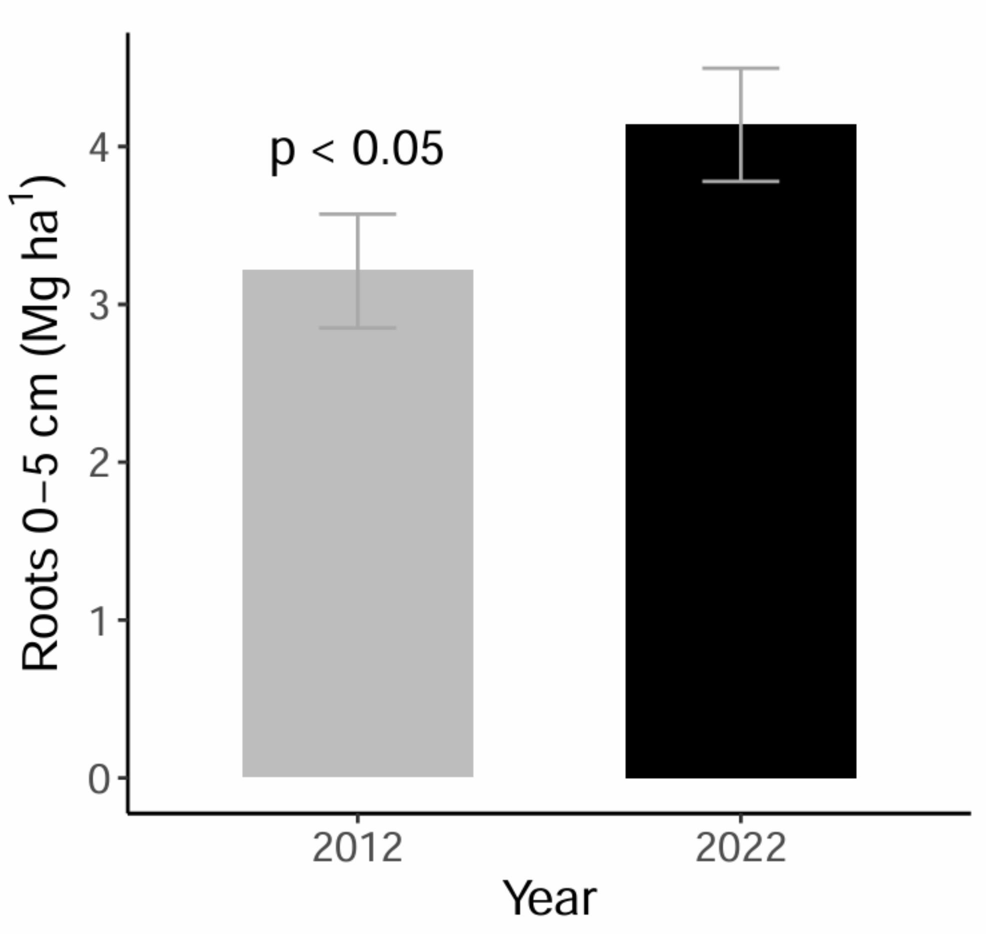

FIGURE S5 Root biomass in the topsoil of European beech stands in the Vienna Woods, Austria, for the sampling years 2012 and 2022 (mean ± SE; *n* = 62).

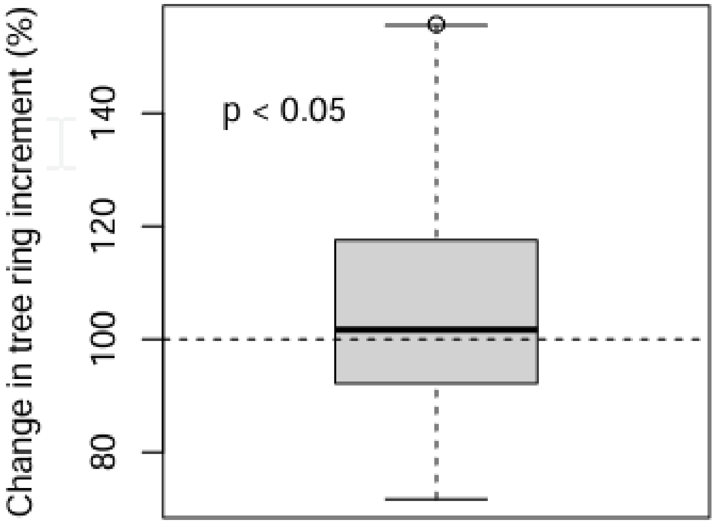

FIGURE S6 Change in annual tree ring increment comparing the growth period between 1984 and 2022 to the period between 1950 and 1984. Tree ring increment data were corrected for age-related trends prior to analysis.

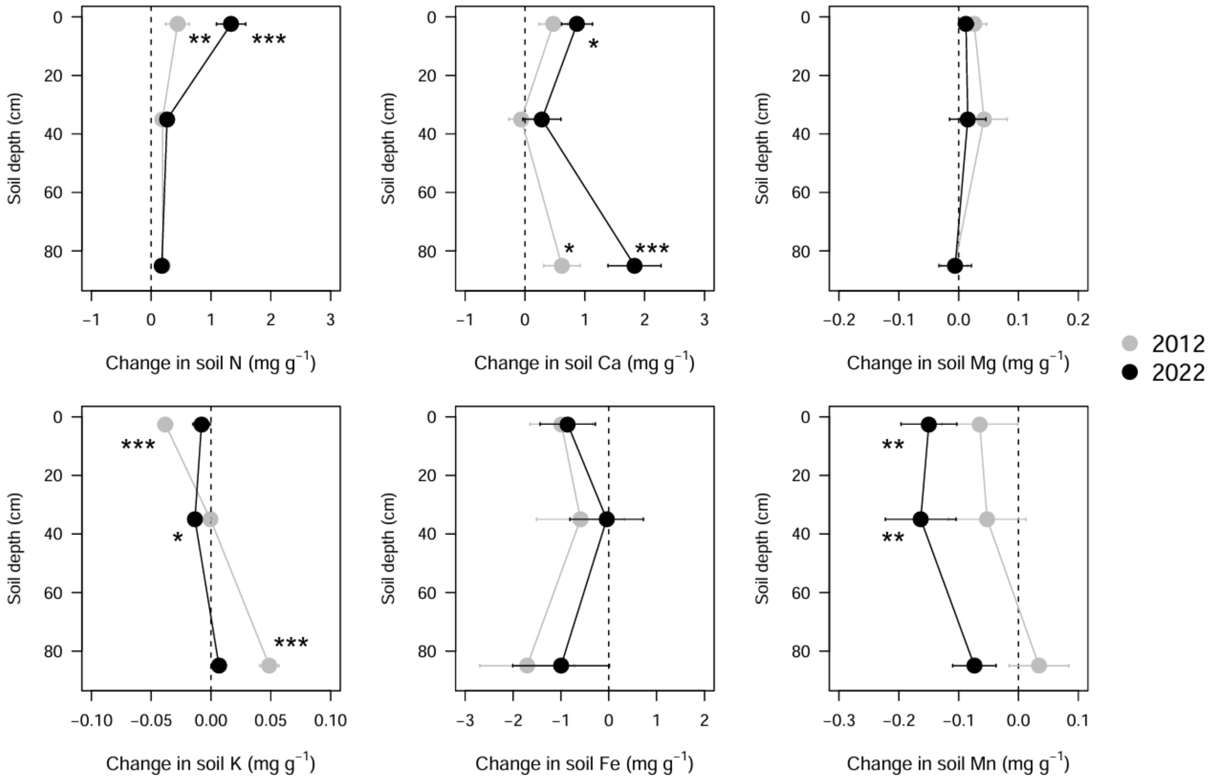

FIGURE S7 Changes in total soil nitrogen (N), exch. calcium (Ca), exch, magnesium (Mg), exch. potassium (K), total iron (Fe), and total manganese (Mn) content at three depths of European beech stands in the Vienna Woods, Austria, in 2012 and 2022 as compared to 1984 (mean ± SE; n = 62). Significant differences from zero are indicated by asterisks (***$p < 0.001$; **$p < 0.01$; *$p < 0.05$).

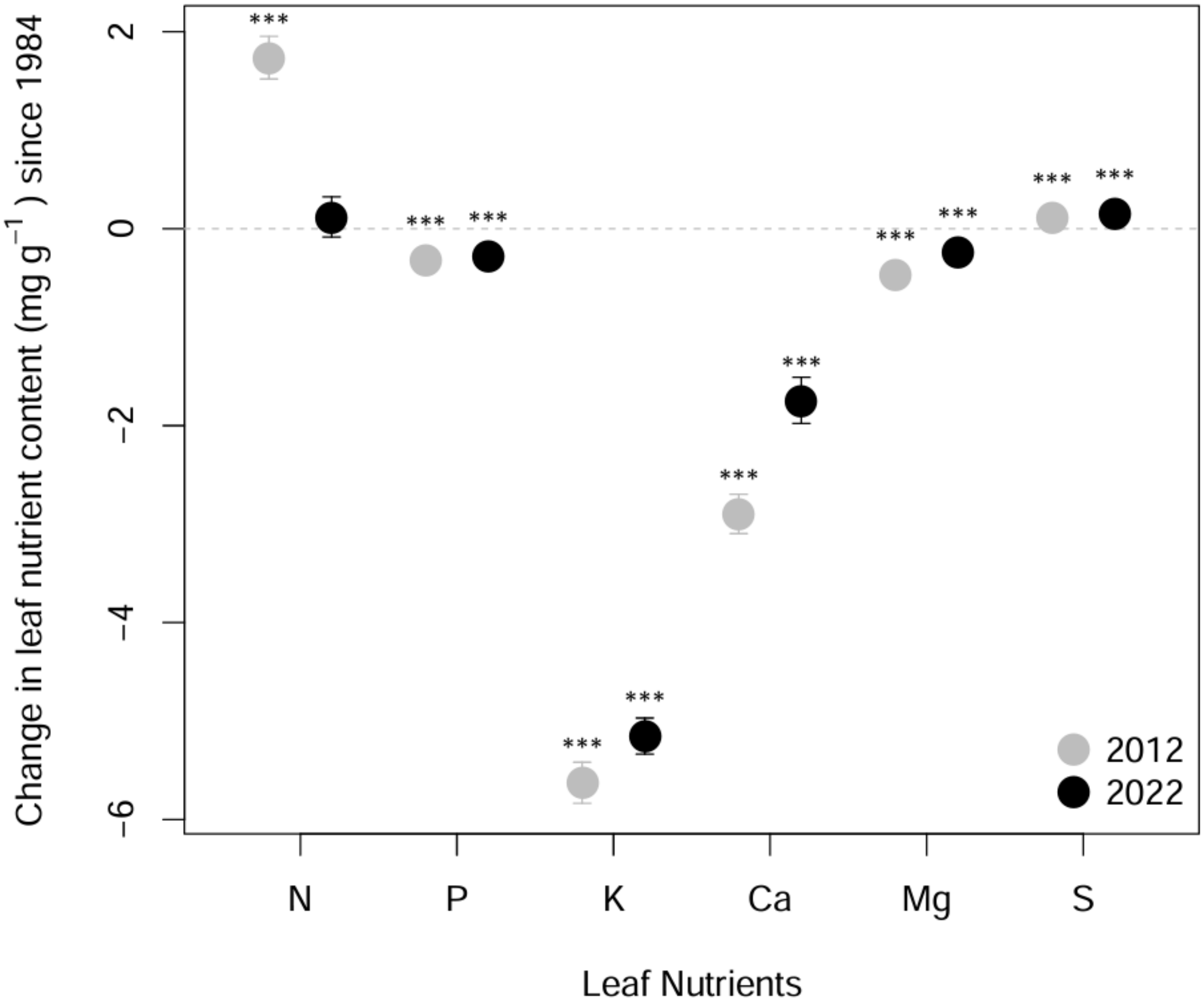

FIGURE S8 Changes in leaf nitrogen (N), phosphorus (P), potassium (K), calcium (Ca), magnesium (Mg), and sulfur (S) content of European beech stands in the Vienna Woods, Austria, in 2012 and 2022 as compared to 1984 (mean ± SE; n = 62). Significant differences from zero are indicated by asterisks (***$p < 0.001$).

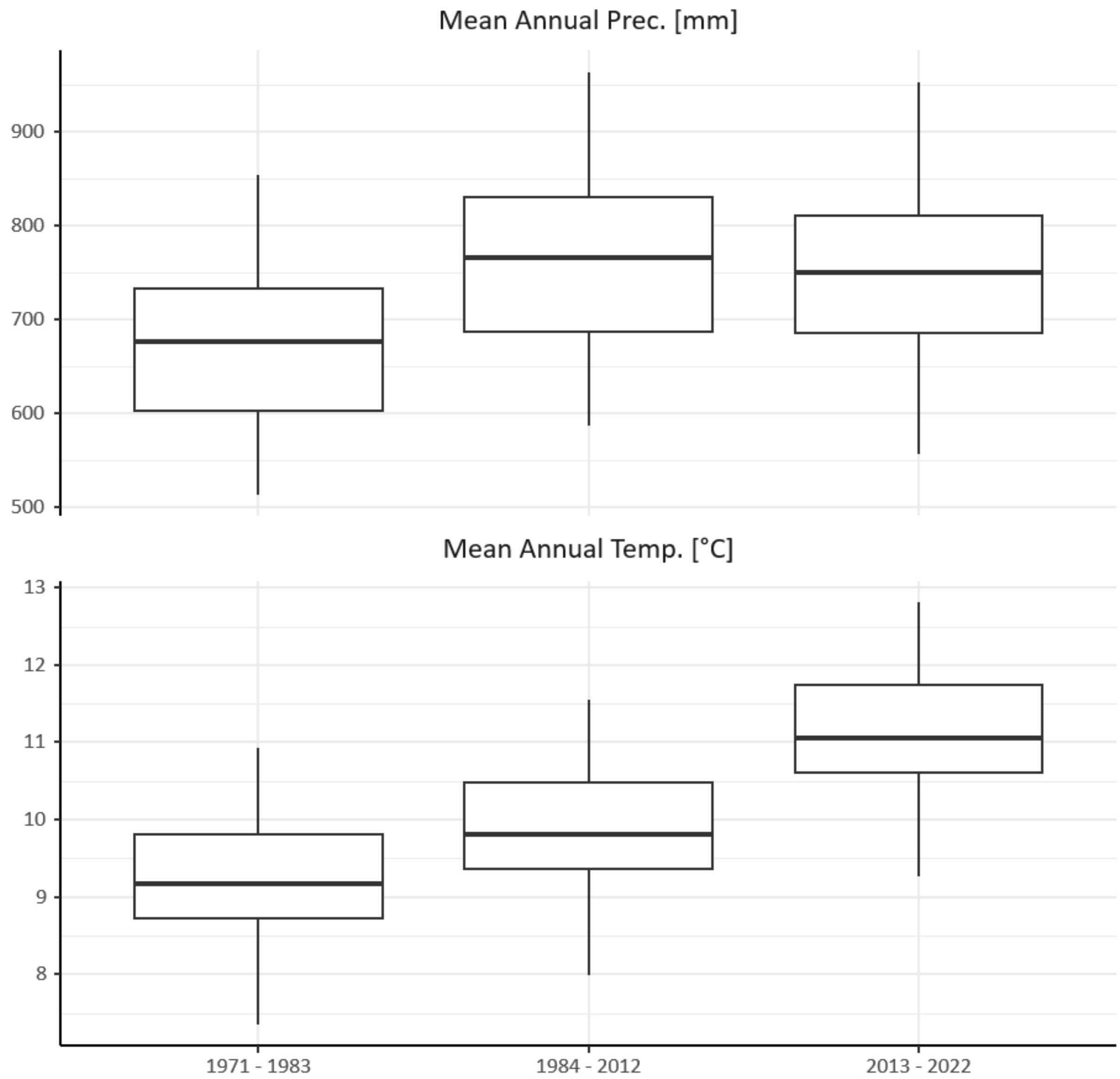

FIGURE S9 Boxplots showing the median, along with the distribution, of mean annual precipitation and mean annual temperature for 62 European beech stands in the Vienna Woods, Austria, across three periods: before and between the sampling years 1984, 2012, and 2022. Data are derived from Geosphere Spartacus (Hiebl & Frei, 2016).
